# Evaluation of Pelvic Floor Muscles in Pregnancy and Postpartum With Non-Invasive Magnetomyography

**DOI:** 10.1109/JTEHM.2021.3130785

**Published:** 2021-11-25

**Authors:** D. Escalona-Vargas, E. R. Siegel, S. Oliphant, H. Eswaran

**Affiliations:** Department of Obstetrics and GynecologyUniversity of Arkansas for Medical Sciences 12215 Little Rock AR 72205 USA; Department of BiostatisticsUniversity of Arkansas for Medical Sciences 12215 Little Rock AR 72205 USA

**Keywords:** Biomagnetism, levator ani muscles, magnetomyography, pregnancy, pelvic floor

## Abstract

*Objective*: To record and characterize features of levator ani muscles (LAM) activity in pregnancy and postpartum using non-invasive and novel Magnetomyography (MMG) technique with amplitude and spectral parameters. *Methods*: Nulliparous women with uncomplicated pregnancies participated in the MMG data collection during rest and voluntary LAM contractions (Kegels) with modulated intensity in third trimester and approximately 2 months postpartum (PP). Simultaneous surface electromyography was recorded to document the recruitment of accessory muscles. Moderate strength Kegel (MK) MMG trials were analyzed. Amplitude and spectral parameters including root-mean square (RMS) amplitude, power spectrum density (PSD) and normalized PSD (rPSD) in three frequency bands (low, middle, high) were computed on MK epochs. Statistical comparisons between pregnancy and postpartum were calculated. *Results*: MMG recordings were measured from 10 pregnant women. Results showed decreased RMS and power between third trimester and postpartum, trending towards significance. rPSD values in the low frequency band decreased significantly (p = 0.028) from third trimester to postpartum, while significant increase was observed in the middle frequency band (p = 0.018). *Conclusions*: This study shows that MMG as non-invasive tool has the ability to detect and characterize changes of LAM activity with amplitude and spectral parameters during pregnancy and postpartum.

## I. Introduction

The levator ani muscles (LAM) are integral to pelvic floor support and injury to this muscle complex has been associated with pelvic floor disorders. The LAMs provide structural support to the pelvic organs, maintaining continence of bladder and bowel, and support of the vagina and uterus [1]. Human parturition is traumatic to the maternal pelvic floor, with the LAMs undergoing significantly more stretch at vaginal birth than skeletal muscle can normally accommodate without profound injury [2]. It is likely that term vaginal birth causes nearly universal micro-injury to the LAM, with up to 40% of women experiencing clinically significant injury. This injury is often the inciting event in a causal pathway leading to the development of female pelvic floor disorders (PFDs), including pelvic organ prolapse, urinary incontinence, and fecal incontinence, which affect up to 25% of adult women [3].

Animal models have demonstrated that skeletal muscle injury occurs with stretch ratios of 50–60% from baseline [4]. Simulation models in humans suggest that delivery of a term, molded fetal head requires a stretch of 50–200% greater than that of baseline; thus, near-universal injury would be expected to occur [5]. Stretch injury may also occur to the pudendal nerve during parturition, directly impacting the innervation to the LAMs, leading to poor muscle function and muscular atrophy [6].

Clinically detectable rates of postpartum LAM injury range from 5–40% and vary significantly by risk group and detection method. It is plausible that all primiparous women (women experiencing first pregnancy) sustain at least some degree of micro-trauma to the LAMs, given that <10% of women maintain an intact perineum following first vaginal delivery [7]. Past studies that applied imaging techniques have shown that LAM edema after delivery seems to resolve in many women in the later postpartum period [8], [9]. To what degree this micro-injury is truly recoverable remains unclear. There is a critical void in our understanding of the adaptations and injury recovery patterns of the maternal LAMs.

Prior work exploring LAM injury has used anatomic imaging - magnetic resonance imaging (MRI), ultrasound, functional assessment – electromyography (EMG), and clinical strength measures [10], [11], but none of these modalities offers a complete assessment of LAM function in a temporal and spatial dimension. Recently, a protocol was developed using magnetomyography (MMG) to detect changes of LAM function in nulligravidas (women without prior pregnancy) [12], [13]. MMG is a non-invasive technique to passively record the electromagnetic fields produced by depolarization of muscular activity and provides a functional view of muscle activity patterns [14]. The goal of this work was to utilize the novel technology of MMG to noninvasively measure and characterize LAM activities in pregnancy and postpartum.

## II. Methods

### A. Subjects and Measurements

We enrolled a convenience sample of 10 nulliparous women (women that is pregnant currently, and no prior deliveries) for this study in 3rd trimester, and within 2 months postpartum (PPT). None of the pregnant woman had any identified neurologic condition, connective tissue disorder, or vaginal prolapse at time of study enrollment.

**FIGURE 1.**
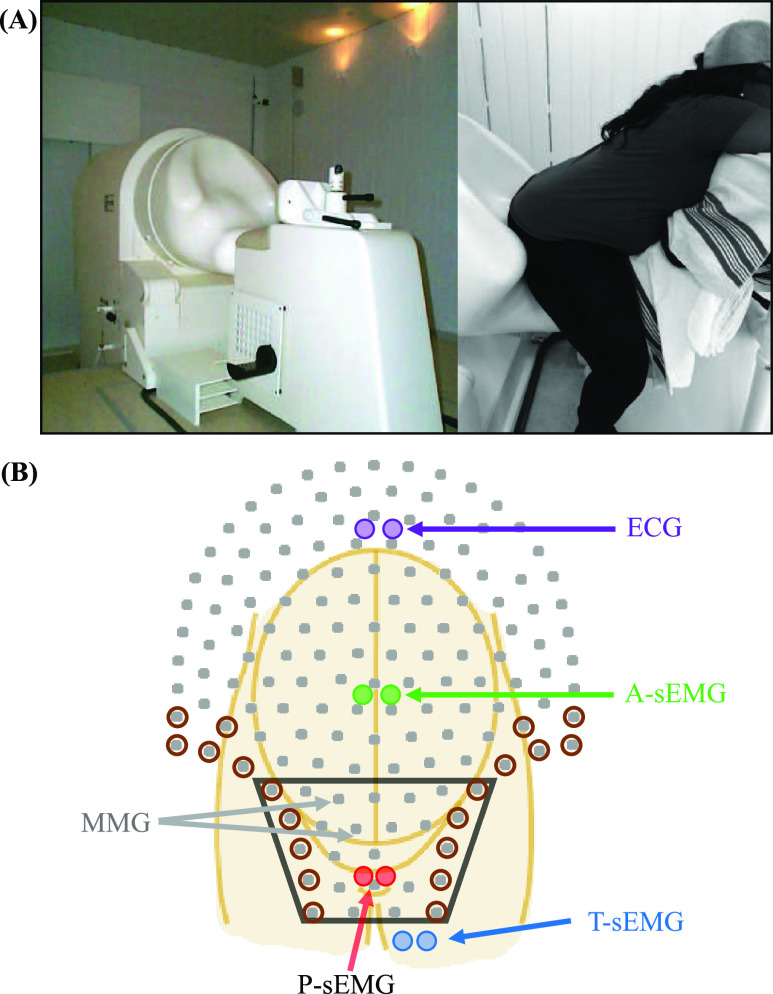
(A) View of the SARA system with the participant positioned for recording. (B) SARA sensor array is represented by grey dots; sEMG abdominal electrodes (labeled as A) by green dots; perineum electrodes (labeled as P) by red dots and thigh electrodes (labeled as T) by blue dots; maternal ECG electrodes by purple dots. Lower MMG sensors are highlighted. Reference sensors to attenuate thigh activities for SUBTR technique are presented by brown circles.

MMG data was acquired using the 151-sensor SARA (Superconducting Quantum Interference (SQUID) Array for Reproductive Assessment) system (Fig. 1) during voluntary contractions of the LAM (Kegels) with intervals of rest. Recordings were collected under the protocols approved by the Institutional Review Board and participants provided informed and written consent. Data was collected with similar protocol as our previous work in nulligravidas [12], [13] with a sampling rate of 1200Hz. In brief, each pregnant woman was instructed to perform voluntary LAM contractions (Kegels) generating subjective small (SmK), moderate (MK), and strong (StK) Kegels. We encouraged the subjects to perform Kegels with 10s duration with intervening 10s rest. Further, 10s purposeful isolated abdominal (A) and thigh (T) muscles contractions were measured. Pregnant woman had a transperineal ultrasound and clinical pelvic exam for confirmation of ability to perform Kegel contractions. We utilized simultaneous surface EMG to evaluate for accessory muscle recruitment. EMG electrodes were located on abdomen, thigh, and perineum in bipolar configuration (see Fig. 1B). Additionally, one electrocardiographic (ECG) sensor was positioned on the chest of the mother to further remove cardiac activity artifact. During the recordings, the MMG data was marked for the start and end of exercises as: SmK, MK, StK, A, T. As we found in our previous study in nulligravidas [12], the major interference in the MMG activity data was related to activation of thigh and abdominal muscles. This muscular contamination is commonly noted in pelvic floor muscle evaluations [15]. Thus, we modified our previous measurement protocol [12] by adapting a new maternal positioning, which approximated the pelvic floor muscles of interest more directly over the SARA sensor array. Fig. 1A demonstrates maternal positioning from this modified protocol where the subject was positioned facing away from the SARA device. This new position avoids the signal contamination from abdominal muscle activity and improves the capture of LAM specific activity.

### B. Signal Processing

A previous developed frequency dependent subtraction (SUBTR) filter was applied for attenuation of thigh activities [16]. Next, a band-pass filter between 20–200 Hz (4th order Butterworth) along with a notch filter (60Hz) were applied to the signals. To execute SUBTR, we used as reference signals the channels closest to the thigh muscles (brown circles in Fig. 1B). These reference channels were at the edge of the SARA system where we typically observed the thigh muscle activity. Additionally, in order to attenuate any remaining noise and enhance the signal-to-noise ratio (SNR), we applied the independent component analysis (ICA) technique to the MMG data.

An open-source software (Brainstorm [17]) was used for reviewing the quality of pre-processed MMG data. Only lower sensors of SARA channel array were used for further analysis, as these sensors were positioned closest to the muscles of interest. The clean MMG data was split into epochs in the window ranging from −3s to +10s with respect to the trigger when voluntary contraction started. We considered the 3s of data before the voluntary contraction as baseline which was then used to apply baseline correction to the 10s data, a common practice in EMG studies [18]. For the accompanying EMG data, the same pre-processing methods were applied as described above for MMG and the cardiac artifact was removed using SUBTR with reference data from maternal ECG electrode.

### C. LAM Parameters

The LAM parameters were extracted from MMG epochs. The metrics were calculated using MK epochs in order avoid any confounders that can influence SmK and StK. The rationale for this approach was based on the fact that while SmK can produce weak signals and the StK can be contaminated by artifacts from the use of other accessory muscles, thus affecting accuracy of LAM contraction and strength measurements. Contributions from accessory muscle groups are common in Kegel studies [19]. The signal quality was determined by SNR, which was defined as the square of the ratio between the root-mean square (RMS) amplitude of the signal and the RMS amplitude of the baseline activity (reported in dB). As in previous reports [12], [20], the parameters calculated were: RMS amplitude, power spectral density (PSD) and relative PSD (rPSD). RMS amplitude was calculated using a sliding window approach with 200ms length and 199ms overlap [21]–[23]. Sliding window method is commonly used in kinesiology and motor control studies to low-pass filter the data [23]–[25]. We smoothed the RMS time series via a 100-point moving average. PSD was calculated via Welch’s technique with a sliding window of 1s and 50% overlap. Total power was calculated as the sum of PSD content and used to normalized the PSD (rPSD). rPSD was divided into three frequency bands of 60Hz bins [26]–[28]. Frequency bands were: rPSDL low (20–80Hz), rPSDM middle (80–140Hz), rPSDH high (140–200Hz).

Since there were multiple MK exercises in each recording per subject, we computed grand averages of the amplitude and frequency parameters per subject. Thus each subject had a pair of parameters in 3rd trimester pregnancy and in early postpartum.

### D. Statistical Analysis

We tested for normality using the Shapiro–Wilk test and applied paired Student t-test technique to evaluate the significance of differences in the RMS, Total Power and rPSD parameters between pregnancy and postpartum recordings (alpha = 0.05).

## III. Results

**FIGURE 2.**
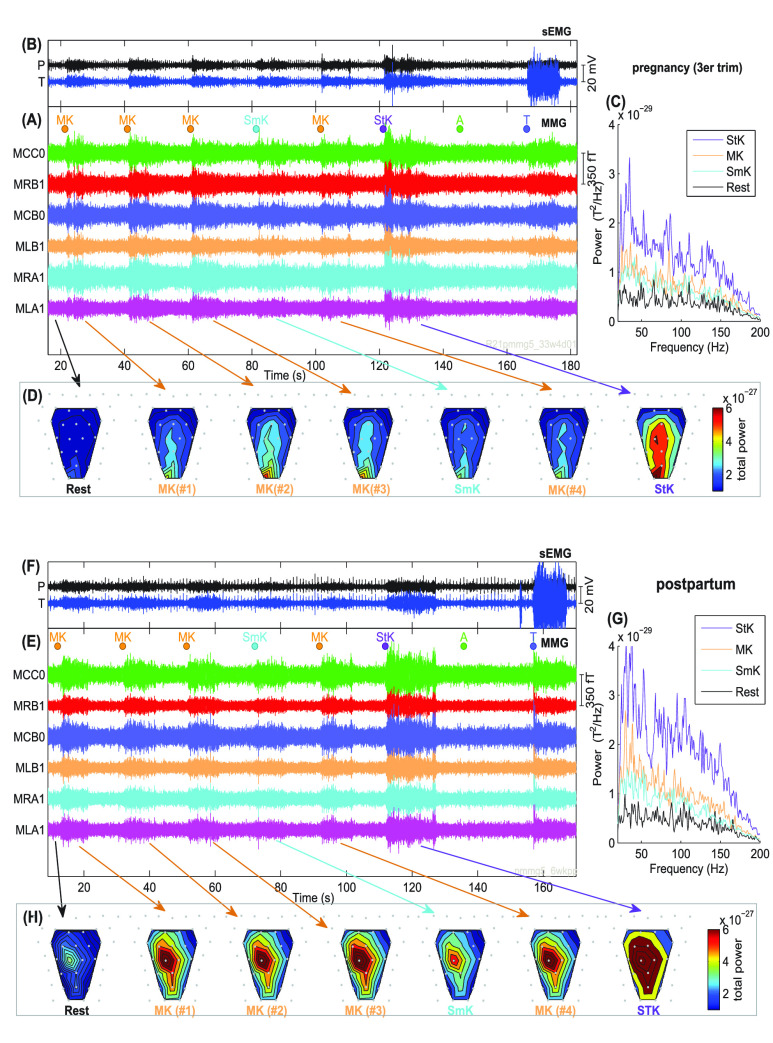
Recordings during pregnancy at GA = 33w4d and postpartum recordings at 6w after delivery. A, E and I: MMG recordings; B, F and G: simultaneous sEMG obtained during rest, voluntary Kegels (Sm = small, M = moderate, St = strong), and abdominal (A) and thigh (T) contractions. Filled circles on the top of figures denote the start of an exercise (10s intervals). sEMG were measured from locations - perineum (P), and thigh (T). The corresponding PSD (panels C, G and K) and total power maps (panels D, H and L) extracted from MMG signals are shown in the figure.

We were able to successfully record LAM signals from 10 pregnant women. The demographic information and participant recording characteristics are shown in Table 1. After excluding MK epochs below threshold SNR < 1dB, we discarded one subject for further analysis because all MK epochs from her postpartum recording did not attain SNR threshold, thus it was not possible to obtain pair of parameters in both pregnancy and postpartum. MMG data of one mother is present in Fig. 2 showing the rest and voluntary muscle contractions periods for her recordings in third trimester and postpartum. Panels show MMG (Fig. 2A and 2E), sEMG (Fig. 2B and 2F) when LAM exercises (SmK, MK, StK), and the A and T exercises. Filled circles above MMG data, show the start of each exercise. Fig. 2 also shows the averaged PSD distributions between 20 to 200Hz (Fig. 2C and 2G) across MMG sensors and the total power maps (Fig. 2D and 2H) for the different Kegels. Maps show the sum of the power across frequencies of the MMG activities per SARA sensor. Power maps are contour plots color-coded with an overlay on the SARA sensor array to show the spatial distribution. As seen in the figure, the contour map reflects spatial distribution and the increase in power value as the participant proceeds through the Kegel sequence starting at rest (blue) and ending in a strong Kegel (red).

**TABLE 1:** Overview of Participant Demographics and Other Characteristics

Maternal Age, years	
Mean (SD)	26.2±3.3
Range	22–31
Pre-pregnancy BMI, kg/m^2^	
Mean (SD)	29.0±6.7
Range	20.6–41.8
Maternal Race, N (%)	
African-American	5 (50%)
White, non-Hispanic	5 (50%)
Delivery Type, N (%)	
Spontaneous vaginal	7 (70%)
Cesarean section	3 (30%)
Gestational Age at Delivery, weeks	
Mean (SD)	38.5 (1.1)
Range	37–40

**FIGURE 3.**
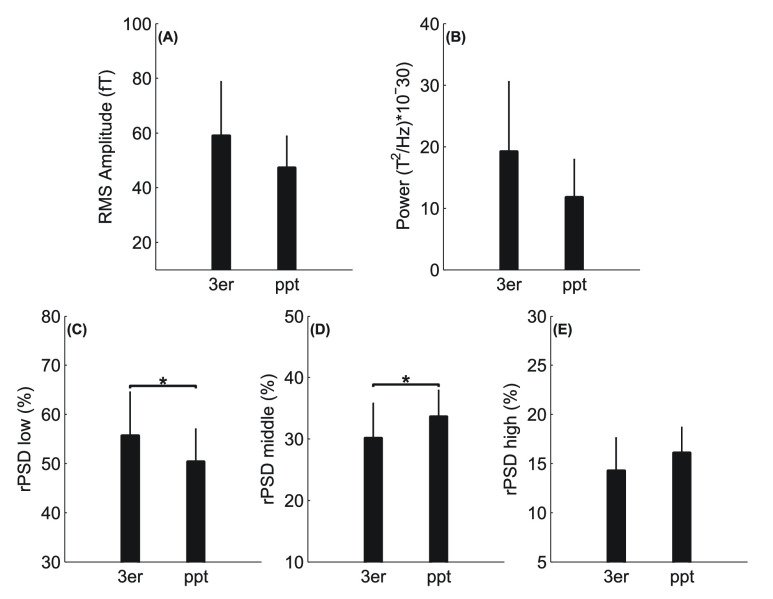
Mean and standard deviation of MMG MK parameters. Statistical analysis was performed via the t-test method. RMS: root-mean square amplitude; PSD: power spectral density; rPSD: normalized PSD into frequency bands: low (20–80Hz), middle (80–140Hz), high (140–200Hz).

The comparative group level analysis of the amplitude and spectral parameters of 3rd trimester and postpartum recordings are shown in Table 2 and Fig. 3. Paired analysis shown a decrease in RMS amplitude and power between 3rd trimester and postpartum, trending towards significance. While significant decrease in values of rPSD in the low frequency band (p = 0.028) from 3rd trimester to postpartum were observed, a significant increase was observed in the middle frequency band (p = 0.018).

**TABLE 2:** Paired-Data Analysis (Third Trimester, Postpartum). Values (Mean±SD) of RMS, Total Power, and rPSD at Third Trimester (3er) and Postpartum (PPT). Only Nine Subjects Were Included in the Analysis Because MK Epochs From One of the Subjects From the Postpartum Session was Excluded Due to Low SNR

Metric	3er	PPT	Delta	SD(Delta)	p-val
RMS	58.9±19.9	47.2±11.5	−11.69	15.8	0.0578
Power	19.2±11.3	11.7±6.2	−7.46	9.82	0.0521
rPSD low	55.6±8.91	50.3±6.7	−5.31	5.98	0.0287
rPSD middle	30.1±5.6	33.6±4.3	3.46	3.54	0.0189
rPSD high	14.2±3.4	16.1±2.6	1.85	2.54	0.0604

## IV. Discussion

Based on our knowledge this the first non-invasive study that uses MMG technique to record and characterize LAM activities to assess its function during pregnancy and postpartum at the electrophysiological level. This study showed postpartum RMS MK amplitude values were lower than in pregnancy, and shifts of power values (i.e. decrease in the low frequency band of PSD) occurred from pregnancy to postpartum. The only other non-invasive modality that captures functional information is the surface EMG [29]; although it reflects only the secondary currents reaching the skin surface and it has well-known limitations [30]. Thus, some of the EMG basic characteristics can be compared to our MMG data. Pereira et. al [31] characterized the LAM activities with surface EMG signals across different phases of the female lifecycle. Based on their study, higher EMG amplitude was observed in nulliparous women than any other group studied, including pregnant, postpartum, and postmenopausal women. Similarly, the MK mean RMS and power reported on nulligravidas (see reported values in previous work [12] on Table 1; RMS: 120.66 ± 43.8 fT, and power: 1.72 ± 1.44 ( $T^{2}/Hz)*10^{-28}$) in an earlier MMG study was much higher than what we observed in the current study on nulliparous women (see Table 2). Another study by Botehlo et. al [32] on 75 primiparous women, compared the pelvic floor EMG measures during the third trimester of pregnancy and 45 days postpartum. Authors showed that mean RMS values were significantly lower in maximum contraction of the LAM after vaginal delivery. Again our observation of pregnancy versus postpartum comparison show higher mean MMG RMS values during third trimester with respect to postpartum. We believe that the decrease in LAM strength seen in pregnant women compared to nulligravid women may be representative of the extensive connective tissue remodeling and changes in tissue distensibility which occurs in the pelvic floor during pregnancy [29], [33], [34]. Further studies are needed to explore this hypothesis. Both of EMG based studies mentioned above are in line with the characteristics extracted from the MMG technique and mirror the amplitude changes observed in LAM between pregnancy and postpartum. However, MMG technique allows to capture undisrupted signals compared to surface EMG where electrical fields are attenuated by the various layers of tissue between the source and the skin surface. While this study has shown that MMG recordings exhibit similar characteristics as EMG, in future works the spatiotemporal resolution of MMG can offer unique characteristics of LAM to complement and increase our understanding of maternal adaptation during pregnancy and postpartum. With our adapted protocol for MMG LAM assessment in pregnancy, we plan in future work to recruit a larger and more diverse population to further characterized maternal LAMs adaptation during pregnancy and explore the identification of patters of LAM injury and recovery to ultimately identify pregnant women at highest risk. Further, we plan to investigate additional signal parameters extracted from MMG activities that typically are used in skeletal muscles studies [25] including a more detail study to explore if specific frequency bands of the MMG signals are more susceptible to alteration in pregnant women with LAM disorders.

Although the limitation of this study is its small sample size, we were able to demonstrate an excellent ability of MMG to characterize LAM function during pregnancy and postpartum. Due to the limited sample size, we did not include maternal characteristics such as mode of delivery, body mass index and maternal age, in our analysis. In our future prospective study, we will plan to correlate all these variables with MMG data. We recognize that some subjects may be unable to volitionally contract their LAM and perform an adequate Kegel, which could impact our ability to collect accurate MMG measures, but with our newly modified protocol, we were able to capture signals with enough SNR. As mentioned earlier, since our study represented the initial foray to translate the unique MMG technique to measure LAM activities, some parameters in this study were based on well-established EMG studies, while others were exploratory. The sampling rate was based on what is normally used for skeletal muscles, around 1 KHz, which also satisfied the Nyquist criteria required for pelvic floor muscle studies. Further, amplitude measures presented here including the RMS values were commonly reported in pelvic floor EMG studies, while the different time and frequency parameters were exploratory. There is no prior work that included frequency domain and splitting the frequency content of the signals, such as frequency bands splitting, the influence of contraction type, and LAM strength, but will be included in future studies for improved characterization of MMG signals. Due to the current cost of MMG technology, this technique is best suited for research endeavors but does provide a unique opportunity to improve our knowledge of the electrophysiology of the female LAM during pregnancy. We hope that current advances in biomagnetic technology [35] will soon allow us to collect MMG data using a smaller footprint, with less expense and a flexible array of sensors, and in future translate the MMG modality into direct clinical applications. Finally, MMG can be applied to other settings including detection of LAM dysfunction in women with pelvic organ prolapse or with pelvic floor hypertone in patients with deep infiltrating endometriosis [36].

## V. Conclusion

Our work demonstrates the ability of MMG technique to capture LAM contraction during pregnancy and postpartum. The MMG modality provides a non-invasive and novel approach to assess female LAM function. In this work in nulliparous women, we characterized changes of LAM activities from pregnancy to postpartum using time and frequency parameters. Our work adapts biomagnetic technology to advance our knowledge regarding maternal muscular adaptations and maternal LAM injury. We expect that rapid advancements in biomagnetic technology, including a less expensive and more flexible array of low-cost and low-maintenance biomagnetic sensors, will aid in clinical translation and wider adaptation of MMG technology.

## Conflict of Interest

The Authors declare that there is no conflict of interest.
